# From vestibular implant to cortex: electrically evoked vestibular responses

**DOI:** 10.1007/s00415-025-13158-1

**Published:** 2025-05-29

**Authors:** Stan C. J. van Boxel, Joost J. A. Stultiens, Marc van Hoof, Bernd L. Vermorken, Benjamin Volpe, Nils Guinand, Angélica Perez-Fornos, Erik D. Gommer, Elke M. J. Devocht, Andreas Zwergal, Marcus L. F. Janssen, Raymond van de Berg

**Affiliations:** 1 Department of Otorhinolaryngology and Head and Neck Surgery, Division of Vestibular Disorders, Maastricht University Medical Center, Maastricht, The Netherlands; 2 Mental Health and Neuroscience Research Institute (MHeNs), Maastricht University, Maastricht, The Netherlands; 3 German Center for Vertigo and Balance Disorders (DSGZ), LMU University Hospital, Ludwig-Maximilians-Universität München, Munich, Germany; 4 Department of Neurology, LMU University Hospital, Munich, Germany; 5 Service of Otorhinolaryngology Head and Neck Surgery, Department of Clinical Neurosciences, Geneva University Hospitals, Geneva, Switzerland; 6 Department of Clinical Neurophysiology, Maastricht University Medical Center, Maastricht, The Netherlands

**Keywords:** Vestibular brainstem response, Vestibular implant, Vestibular cortex, Electroencephalography, Evoked potentials

## Abstract

**Purpose:**

Understanding central vestibular pathways remains challenging and requires innovative measurement approaches. A vestibular implant offers unique access through specific electrical stimulation of the vestibular end organ. This study explored the feasibility of using vestibular implant stimulation to obtain vestibular evoked potentials, using electroencephalography (EEG).

**Methods:**

A vestibular implant was used in nine participants to evoke vestibular potentials by targeting the ampullary nerves of the semicircular canals. Short latency potentials were recorded using one channel EEG on all participants. In three participants, long latency potentials were recorded with 128 channel EEG. Responses were analyzed in terms of latency, shape, and location, and tested for correlation with stimulus intensity. EEG thresholds were compared with vestibular outcome thresholds (i.e., perception and vestibulo-ocular reflexes).

**Results:**

The measurement setup proved feasible for obtaining vestibular potentials. A consistent short latency response, identified as the vestibular brainstem response, was identified in five participants and across targeted nerves. Long latency responses revealed defined and localized independent components, with amplitudes correlating with stimulus intensity. Electrically evoked response thresholds matched thresholds of patient perception and eye movement recordings.

**Conclusions:**

Vestibular implant stimulation elicited reproducible short and long latency responses. This approach creates new opportunities for investigating vestibular processing and evaluating vestibular implant responses.

**Supplementary Information:**

The online version contains supplementary material available at 10.1007/s00415-025-13158-1.

## Introduction

The vestibular organ is part of a multisensory system crucial for gaze stabilization, postural control, and spatial orientation [1]. Understanding vestibular disorders necessitates a thorough knowledge of normal vestibular neurophysiology. To date, however, the exact functioning of the vestibular neural network from the inner ear to the higher-order cortical brain regions remains incompletely elucidated [2–8].

To study neural processing, several measurement setups have been developed, all with their own advantages and disadvantages in stimulation and measurement methods [9–13].

The development of investigational vestibular implants offers a unique opportunity to this field. This artificial balance organ offers direct electrical stimulation of the most peripheral nerve afferents, at the individual semicircular canal [14]. The stimulation is highly controllable and localized, offering exceptional temporal resolution and resulting in robust responses (e.g., vestibulo-ocular reflex, movement perception) [15]. The advantages of using a vestibular implant can be enhanced by incorporating electrophysiological measurements.

In clinical audiology, the short latency auditory brainstem response (ABR) serves as a key measure for objectively assessing auditory thresholds and auditory nerve and brainstem function [16]. In contrast, the vestibular equivalent is not conclusively characterized yet and has not yet been adopted in clinical practice (reviewed in [10] and [9]). The current measurement setup remains burdensome and complicated, with substantial limitations and minimal clinical implications. Vestibular implants, however, provide the opportunity to evoke responses in a setup equivalent to the well-known cochlear implant setup of electrically evoked ABR (eABR) [16]. Using this approach, a short latency response, proposed as the vestibular brainstem response (VBR), could be characterized. Next to this short latency response, neural processing can be followed up to the cortical level. Studying long latency responses enables identification of the brain areas involved in higher-order processing of vestibular information.

The objective of the study is to improve understanding of the vestibulo-brainstem-cortical network, by studying electrically evoked vestibular potentials. Furthermore, response characteristics were analyzed and compared with stimulation amplitude and vestibular outcome thresholds (i.e., perception and vestibulo-ocular reflexes).

## Materials and methods

### Participants

This study was conducted as part of the VertiGo!-trial (ClinicalTrials.gov Identifier: NCT04918745). Patients with bilateral vestibulopathy and severe sensorineural hearing loss were included (Table 1). Information related to inclusion, surgery and implant can be found in the trial protocol [17].

**Table 1:** Participants characteristics

Participant ID	Sex	Age at implantation (years)	Etiology of vestibulopathy	Duration of symptoms (years)	Year of implantation	Implanted side
VCI-1	Female	54	DFNA-9	7	2021	R
VCI-2	Male	65	Auto-immune (CREST)	21	2021	R
VCI-3	Male	52	DFNA-9	30	2022	L
VCI-4	Male	66	DFNA-9	10	2022	R
VCI-5	Male	28	Idiopathic	4	2022	R
VCI-6	Male	66	M. Meniere	25	2022	R
VCI-7	Female	62	DFNA-9	6	2022	L
VCI-8	Male	63	Skull base fracture	< 1	2023	R
VCI-9	Female	62	Skull base fracture	< 1	2023	R

*DFNA9* autosomal dominant non-syndromic sensorineural deafness 9

The trial was designed in accordance with the declaration of Helsinki. The protocol was approved by and carried out in accordance with the recommendations of the local ethics committee (Maastricht University Medical Center, NL73492.068.20/METC 20-087).

### Vestibular implant

Participants were implanted with an investigational vestibulo-cochlear implant (provided by MED-EL, Innsbruck, Austria). It consisted of three vestibular electrode leads (one contact each), inserted into the ampullae of the three semicircular canals, and an electrode array with nine electrodes inserted in the cochlea (coverage 24 mm). Vestibular target nerves were the lateral, superior, and posterior ampullary nerve (LAN, SAN, PAN). Pre- and intraoperative imaging was used to optimize and verify electrode placement within 1.5 mm of the ampulla [18].

### Study setup

Short and long latency recordings were conducted in separate experiments. Short latency recordings were acquired from all participants. Furthermore, perceptual and vestibulo-ocular reflex thresholds were obtained (Supplementary Material Sect. 1). The three participants with the strongest vestibulo-ocular reflexes and movement percepts were selected for long latency recording. Responses were evoked from each vestibular electrode independently.

#### Vestibular stimulation

Electrical vestibular stimulation was delivered via the participant’s vestibular implant, utilizing the cochlear implant fitting software and control interface (Maestro, version 9, and MAX-box, MED-EL, Innsbruck, Austria). Stimulation commands were sent via a radiofrequency coil. Electrical stimulation consisted of pulse trains of alternating, biphasic, rectangular, charge balanced pulses, with a phase duration of 200 µs.

For short latency recordings, single biphasic pulses were applied, at a repetition rate of 34 Hz. The stimulation amplitude started at 100 cu (current units; 1 cu ~ 1 mA). After each series, the amplitude was increased with steps of 100 cu until the upper comfortable limit was reached. Stimulation amplitudes were measured twice (each 1500 sweeps). All sweeps from the two series were combined afterward, aiming for a low residual noise level (< 50 nV).

Subsequently, responses were also evoked on one cochlear electrode (number six, where one is the most apical). The morphologies of the vestibular and auditory responses were compared, in order to deduce the origin of the responses. The same parameters as for vestibular stimulation were used.

For long latency recordings, pulse trains with a duration of 10 ms were applied with an intra-burst frequency of 500 Hz, repetition rate of 1 Hz, each with a total of 500 repetitions (based on eABR [16]). Stimulation amplitude was set at the upper comfortable limit and at 50% of this limit.

#### Recording

Short latency responses were recorded using the Eclipse system (Interacoustics, Middelfart, Denmark), designed for measuring auditory brainstem responses. Recording electrodes (silver-chloride) were placed on the contralateral mastoid (inverting), the high forehead (non-inverting), and low forehead (ground). The measurement window was set to start at the onset of stimulation until 25 ms. The sampling frequency was 30 kHz.

Long latency responses were recorded using an EEG system with 128 electrodes (actiCAP, Brain Products GmbH), mounted according to the 10–10 system [19], with a sampling rate of 512 Hz (BrainRT, version 4.03, OSG, Waarloos, Belgium).

### Data processing and analysis

Short latency recordings were analyzed using the Eclipse software. Recordings were filtered (low-pass filter 3 kHz) and averaged (Gaussian weighing). The first 0.5 ms of the recording was excluded from the analysis since it was highly distorted by the stimulation artifact. The residual noise level was calculated and used as indicator for the validity of the obtained response. A higher noise level resulted in a higher risk of masking the response. Response classification and peak detection were performed by an experienced clinical ABR expert, based on visual inspection, the signal-to-noise ratio (cut-off for peak detection SNR 3), and reproducibility (visual and cross-correlation between two buffers).

Long latency recordings were processed using the EEGLAB plug-in for Matlab [20] (MATLAB (R2022b), The MathWorks Inc., Natick, Massachusetts, USA). Noisy channels (root-mean-square signal > 4 times all channel average) were removed and the data were band-pass filtered (0.1–30 Hz) [16]. Data were re-referenced to the common average. Subsequently, independent component analysis (runica; 128 PCA components) was performed [21]. Independent components reflecting eye movements, muscle activity, heart rate, line noise, or channel noise were rejected using IClabel [22]. Only components with an 80% likelihood of originating from brain activity, and with a residual variance < 40%, were kept. Dipole source localization was performed on the components using the DIPFIT source localization module in EEGLAB. Components dominated by an oscillatory response with predominantly 8–12 Hz components originating from the occipital lobe were classified as alpha waves and therefore excluded. Epochs were extracted from −0.05 to 0.4 s relative to the stimulation. Due to the limited sample size and the exploratory nature of the study, data were presented visually and descriptively to illustrate the conceptual outcomes of this novel approach.

## Results

### Short latency response

**Fig. 1:**
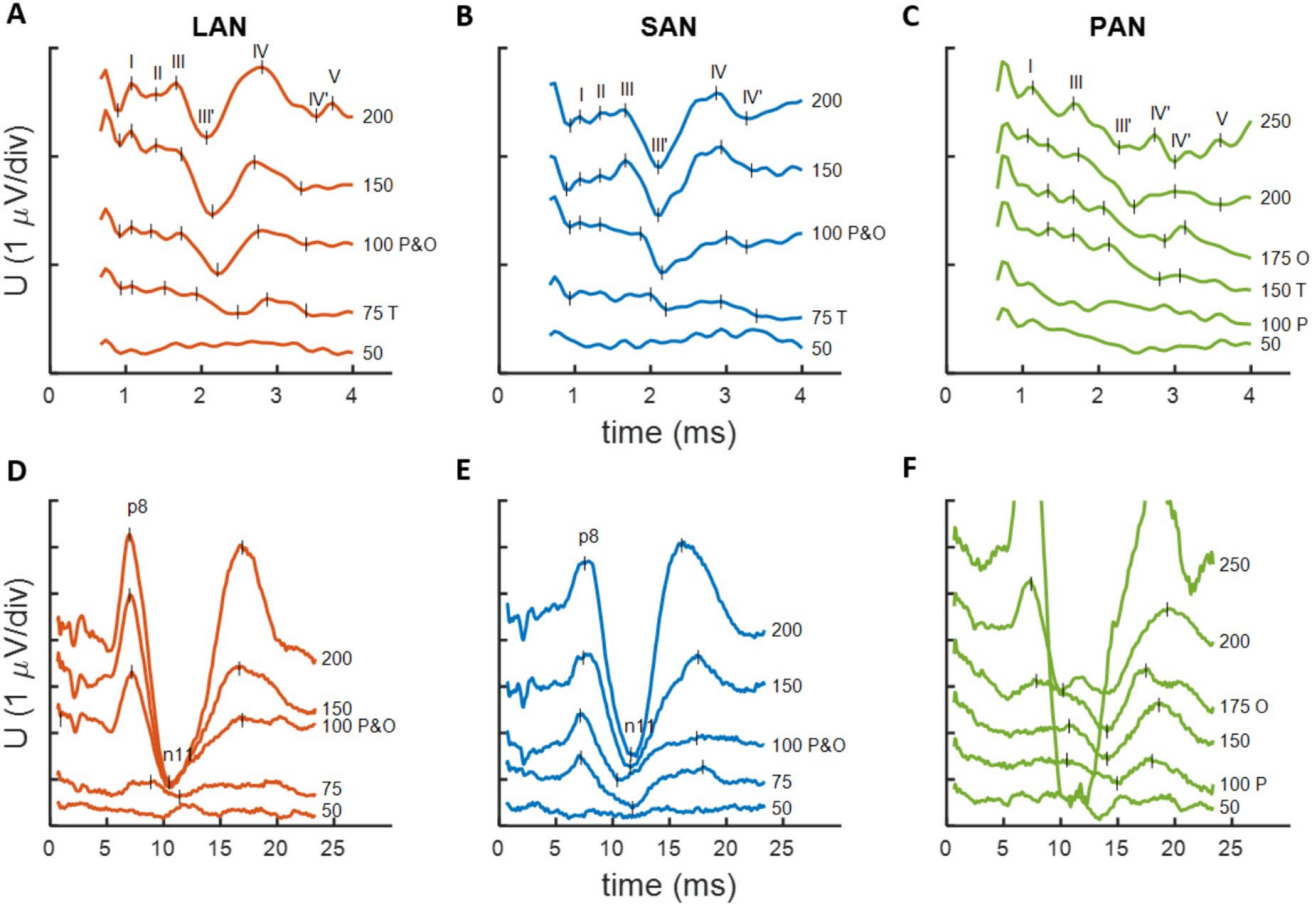
EP recordings of potentials evoked with electrical vestibular stimulation of participant VCI-5. (**A-C)** Displayed traces visualize up to 4 ms post-stimulus. Latin numbers mark the identified peaks and valleys (with apostrophe). (**D-F)** Displayed traces visualize up to 24 ms post-stimulus. P8 and n11 mark the identified positive and negative evoked responses. Responses are shown per stimulation electrode: LAN **(A, D)**, SAN **(B, E),** and PAN **(C, F)**. Values on the right indicate the stimulation level in current units. Vertical markers indicate detected peaks/valleys; “T” indicates the response threshold; “P” indicates the threshold of perception; “O” indicates the threshold of the vestibulo-ocular reflex during stimulation. Abbreviations: EP, evoked potential; LAN lateral ampullary nerve; SAN, superior ampullary nerve; PAN posterior ampullary nerve

Short latency measurements were successfully executed on all participants (Supplementary Material Table 1 and Fig. 1). In seven participants (VCI-1, VCI-2, VCI-5, VCI-6, VCI-7, VCI-8, and VCI-9), similar identifiable responses were obtained. VCI-4 LAN and SAN did not show reproducible responses, despite low residual noise levels. VCI-3 and VCI-4 PAN showed responses with a different morphology. In general, six peaks could be identified (in line with ABR terminology named here as I–V and p8). Not all peaks were visible in all responses. Peak IV and p8 were most distinct and most common. VCI-5 is presented as it serves as an illustrative example of the observed responses (Fig. 1, summarized in Supplementary Material Table 2). These responses showed a peak pattern in the first 4 ms with a smaller amplitude (I–V), and a peak pattern with stronger amplitudes in the 4–24 ms window (p8–n11).

The initial 4 ms of the VCI-5 recordings demonstrated a consistent pattern. The pattern became more discernible with increasing stimulation level. Four peaks (I–IV) were identified, with a distinct III–III’ complex (1.8–3 ms) and a IV–IV’ complex (2.6–3.5 ms). The amplitude of the III–III’ and IV–IV’ complex generally increased, while the latency decreased, with increasing stimulation amplitude.

The first 24 ms of the VCI-5 recordings showed a pattern consistent for all three electrodes, over a range of stimulation levels (Fig. 1D–F). A positive peak appeared around 8 ms (p8), followed by a negative peak around 11 ms (n11). The p8–n11 amplitude increased with increased stimulus amplitude.

Response thresholds (I–V and p8–n11) were within 25 current units of the patient perception and eye response thresholds.

VCI-3 and VCI-4 did not demonstrate I–V responses. Of those, only on the VCI-4 PAN recording the p8–n11 peak appeared. The VCI-3 recordings revealed a positive peak with a latency of around 9 ms.

All but VCI-3 demonstrated a vestibulo-ocular reflex as a result of implant stimulation, and VCI-4 had only minimal vestibulo-ocular reflex responses (peak eye velocity < 4°/s) (Supplementary Material Table 1).

All participants showed a response identifiable as an auditory brainstem response, when stimulating with cochlear electrode six (example of VCI-5 in Supplementary Material Fig. 2). The responses had a different morphology than the vestibular recordings. A recognizable peak V was found, together with a pronounced response with a latency equivalent to the p8–n11 complex in the vestibular recordings. However, the auditory p8–n11 response had opposite polarity.

### Long latency response

**Fig. 2:**
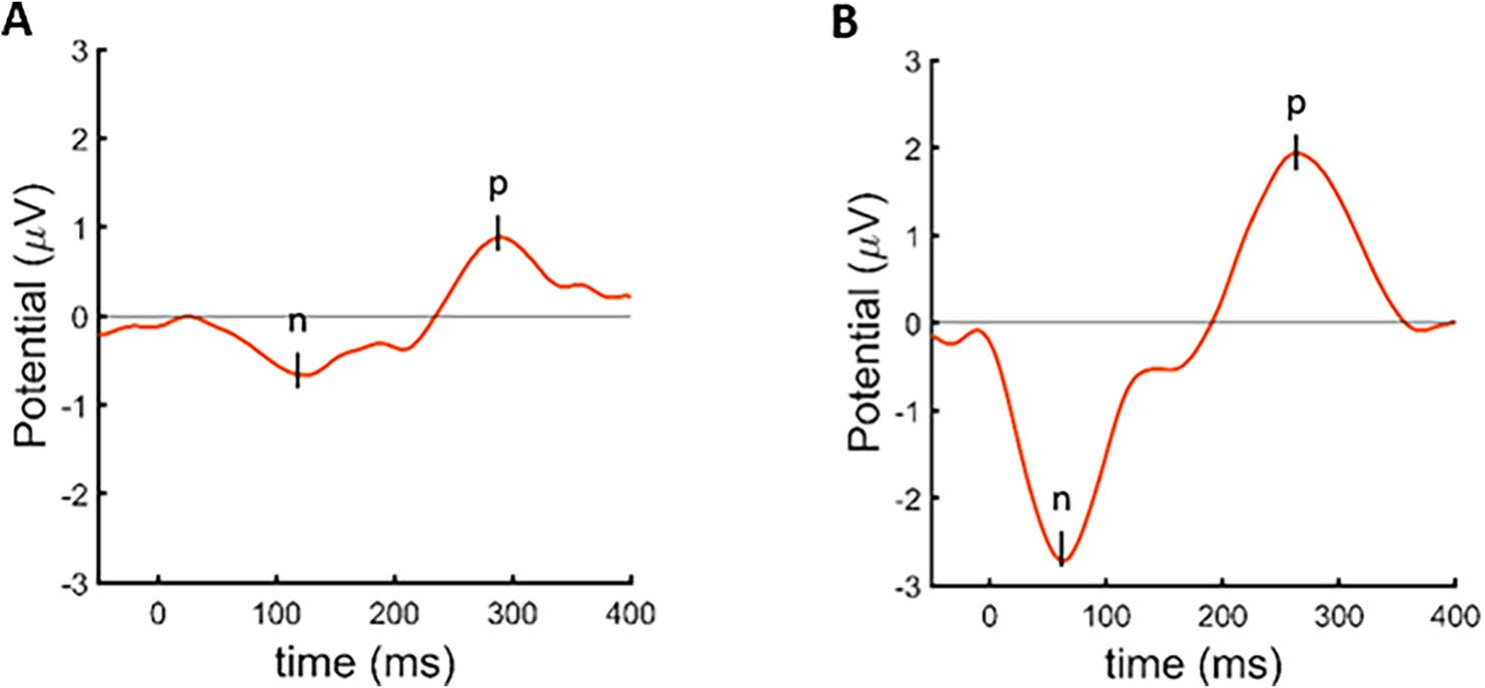
Evoked potentials recorded on the CCP2h electrode, obtained from EEG measurements of potentials evoked with the lateral ampullary nerve electrode of VCI-5. **A** measurements performed with a stimulation level at 50% of the upper comfortable limit and **B** with the stimulation level at the upper comfortable limit. It should be noted that an evoked potential was visible with a negative initial peak, with a latency between 60 and 110 ms (n100). This was followed by a positive peak with a latency of around 270 ms (p270). The amplitude of the response is larger for the higher stimulation level

Three participants (VCI-5, VCI-8, and VCI-9) were selected to assess long latency responses (summary in Supplementary Material Table 3). In all three participants reproducible potentials could be recorded on all three stimulation electrodes for both stimulation levels. Figure 2 shows a representative example from VCI-5 on a central recording electrode (CCP2h). Both recordings demonstrated a clear negative and positive peak (n100, p270). The response at the upper comfortable limit was significantly larger than the response at 50% in all patients and electrodes.

**Fig. 3:**
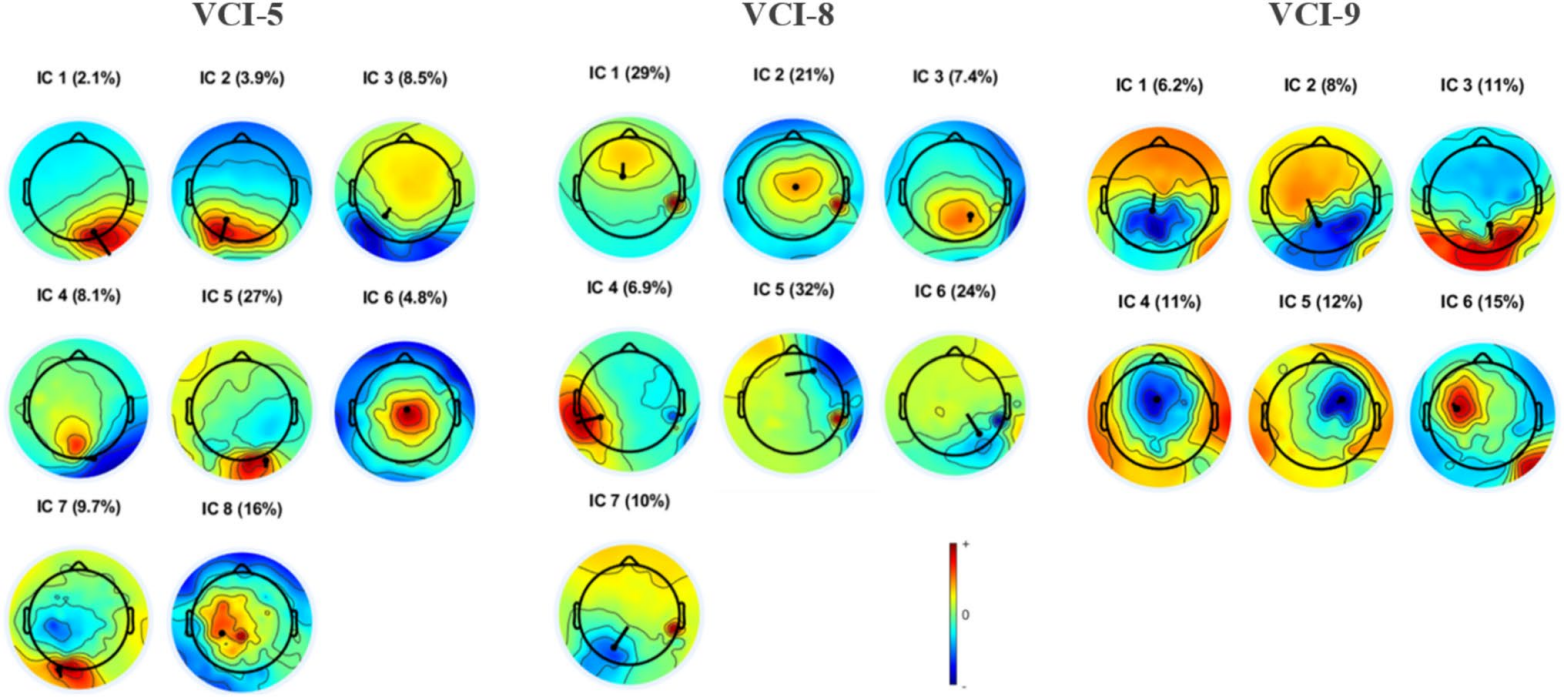
Heat maps and dipoles (black dots with lines indicating direction) of the independent component (IC) maximum activations of three participants (VCI-5, VCI-8, and VCI-9). Percentages indicate the residual variance of the independent component. Three participants were implanted on the right side

The independent component analysis resulted in eight, seven, and six identified independent components for VCI-5, VCI-8, and VCI-9, respectively. Since no differences were found between stimulating electrodes, recordings were combined per participant across stimulating electrodes to increase the number of samples. Heat maps and dipoles visualizing the activity distribution of independent components per participant are displayed in Fig. 3.

## Discussion

This study investigated vestibular evoked brainstem to cortical electrophysiological responses using selective vestibular implant stimulation of the ampullary nerves. Short latency responses were recorded and characterized, classified here as the VBR. Manipulation of stimulus intensity modified the amplitudes and latencies of the peaks of the VBR. Long latency responses, likely originating from higher-order brain regions, were obtained separately. Independent component analyses suggested that the source of late responses originated from different brain regions.

Vestibular evoked potentials with a latency < 10 ms have been associated with the vestibular nerve and nuclei [9, 10, 13]. This implies that the obtained I–V responses are equivalent to the ABR, and can therefore be qualified as the VBR. Latencies might be shorter than reported in literature due to the applied electrical stimulation, which bypasses the first synaptic transmission on the hair cell level [16].

Based on the latency of the responses, together with previous literature, the origin of the neural activation can be hypothesized [10]. Peak I could originate from the vestibular nucleus and II from the contralateral vestibular nucleus (via commissural connection) [23]. Peaks III and IV could represent activity in the ocular motor brainstem nuclei.

Potentials with a latency between 10 and 20 ms have been related to myogenic, cerebellar, or cortical sources [9, 10, 13]. The n8-p11 response was present in all participants who had an electrically evoked vestibulo-ocular reflex. The response was absent in VCI-3 and VCI-4 LAN and SAN, who had either no, or minimal, electrically evoked vestibulo-ocular reflex. Additionally, a different response, having opposite polarity, was present in the recordings when stimulating the cochlear electrodes, possibly originating from the posterior auricular muscle [24]. Lastly, the magnitude of the n8-p11 response, which can reach over 10 µV, also implies a myogenic rather than a neural source. Taken altogether, the response most likely represents the ocular vestibular-evoked myogenic potential, which also is well compatible with the mean latency of the vestibulo-ocular reflex of 8 ms [9].

In line with ABR observations [16], amplitudes of the peaks of the VBR were higher and peak latencies appeared shorter, with higher stimulus intensities. Interestingly, the response thresholds of VCI-5 were within 25 current units of the participant’s perception and eye response thresholds.

Next, a reproducible long latency response including n100 and p270 could be obtained in the three participants included in this analysis. Similar to the short latency responses, the magnitude of the late responses also co-varied with the stimulus level.

Independent component analysis identified different sources contributing to the EEG signal. No clear difference regarding components/dipoles was found between stimulation electrodes (i.e., different ampullary nerves). While selective stimulation of the ampullary nerves was evidently feasible (aside from possible spread of excitation), stimulation of individual channels resulted in the same late responses with the same sources, within participants. This could be a result of early mutual integration of the information coming from the ampullary nerves involving similar brain regions, but potentially different neurons within the same region [25]. Dipole sources of the late responses are largely distributed across the brain and showed heterogeneity among participants (despite same side of implantation). These sources can be associated with brain regions known to be related to vestibular function, such as the parieto-insular vestibular cortex, somatosensory cortex, frontal eye fields, ventral premotor cortex, temporal regions, secondary visual cortex, and higher level visual areas, as well as the thalamus and cerebellum [2–7, 9, 10, 26]. Previous studies with a mastoid galvanic and tone-burst stimulation have also shown a wide-spread arrangement of vestibular cortical potentials with responses in the bilateral anterior insula and posterior operculum at 25–80 ms, in frontal regions at 30–110 ms [27, 28]. Further studying of the sources of the late responses evoked by vestibular implant stimulation might require more data from larger cohort of patients.

Altogether, vestibular implants provide a unique novel method for studying vestibular processing. The approach generated consistent short and long latency responses, enabling the identification of brain regions involved in vestibular processing. This will support mapping of brain regions involved in vestibular functioning. Importantly, the response amplitude co-varied with the stimulation amplitude, as well as functional outcome measures. Consequently, these observations pave the way for new research opportunities related to vestibular implants. This could potentially lead to a role in intraoperative electrode testing and (pediatric) device fitting.

## Supplementary Information

Below is the link to the electronic supplementary material.

Supplementary file1 (DOCX 65 KB)
