# Supplementary material for: From vestibular implant to cortex: electrically evoked vestibular responses
Source: J Neurol. 2025 May 29;272(6):430. doi: 10.1007/s00415-025-13158-1 (PMC12122595; doi:10.1007/s00415-025-13158-1)
Supplement: Supplementary file 1 — Supplementary file1 (DOCX 65 KB) [file 415_2025_13158_MOESM1_ESM.docx]

Supplementary material

# Vestibular thresholds

Patient perception and the vestibulo-ocular reflex were used as functional outcome measures to explore the clinical relevance of the evoked responses. Patient perception was recorded after each short latency measurement series by asking the participant whether the stimulation was felt or not. The presence of an electrically evoked vestibulo-ocular reflex was determined by turning on the same stimulations as in the short latency recordings, and tracking the eyes (VisualEyes goggle system, Interacoustics, Middelfart, Denmark). The presence of a response was determined visually as an eye movement that is time linked with the onset of the stimulation. The threshold was defined as the lowest stimulation amplitude with a vestibulo-ocular reflex. The maximum peak eye velocity (PEV) was determined by stimulation with a two-second block stimulation at the upper comfortable limit.^15^

# Tables and Figures

*Table 1: participant’s short latency response latencies and residual noise level measured at the upper comfortable limit. Peak eye velocity was determined in a separate experiment (see methods section). ‘No response’ indicates that no reproducible response pattern could be obtained. Not available indicates that the measurement was not successfully performed due to technical issues. N.i. (not identifiable), indicates that the peak was not visible in the recording or signal-to-noise ratio was <3.*

| Participant ID | Electrode | I  (ms) | | II  (ms) | | III  (ms) | IV (ms) | | V (ms) | p-n  (ms) | | Residual noise (nV) | Peak eye velocity (degrees/s) |
| --- | --- | --- | --- | --- | --- | --- | --- | --- | --- | --- | --- | --- | --- |
| VCI-1 | LAN | n.i. | | n.i. | | 2.1 | 4.0 | | n.i. | 9.4-11.4 | | >150 | 60 |
|  | SAN | n.i. | | n.i. | | 1.7 | 3.3 | | n.i. | 6.9-8.5 | | >130 | 50 |
|  | PAN | n.i. | | n.i. | | n.i | n.i. | | n.i. | 7.1-8.5 | | >100 | 10 |
|  |  |  | |  | |  |  | |  |  | |  |  |
| VCI-2 | LAN | n.i. | | n.i. | | 2.1 | 4.2 | | n.i. | 9.3-10.7 | | >110 | 170 |
|  | SAN | n.i. | | n.i. | | 1.5 | 2.9 | | 4.9 | 9.0-10.1 | | >70 | 110 |
|  | PAN | n.i. | | n.i | | 1.6 | 2.9 | | n.i. | 9.1-10.6 | | >90 | 30 |
|  |  |  | |  | |  |  | |  |  | |  |  |
| VCI-3 | LAN | No response | | | | | | | | | | >60 | 0 |
|  | SAN | No response | | | | | | | | | | >70 | 0 |
|  | PAN | No response | | | | | | | | | | >70 | 0 |
|  |  |  | |  | |  |  | |  |  | |  |  |
| VCI-4 | LAN | No response | | | | | | | | | | >50 | 3 |
|  | SAN | No response | | | | | | | | | | >50 | 2 |
|  | PAN |  |  | | No response | | |  | | | 8.9-12.4 | >40 | 3 |
|  |  |  | |  | |  |  | |  |  | |  |  |
| VCI-5 | LAN | 1.0 | | 1.3 | | 1.7 | 2.7 | | n.i. | 7.1-10.6 | | >40 | 160 |
|  | SAN | n.i. | | 1.4 | | 1.7 | 2.9 | | n.i. | 7.8-11.7 | | >40 | 220 |
|  | PAN | 0.87 | | 1.3 | | 1.8 | 3.1 | | n.i. | 7.0-10.8 | | >40 | 80 |
|  |  |  | |  | |  |  | |  |  | |  |  |
| VCI-6 | LAN | n.i. | | 1.1 | | n.i. | 3.7 | | n.i. | 7.1-9.3 | | >130 | 100 |
|  | SAN | n.i. | | 1.2 | | n.i. | 3.7 | | n.i. | 6.5-9.7 | | >100 | 50 |
|  | PAN | n.i. | | n.i. | | 1.6 | 2.9 | | n.i. | 6.9-8.7 | | >170 | 50 |
|  |  |  | |  | |  |  | |  |  | |  |  |
| VCI-7 | LAN | 0.7 | | 1.3 | | n.i. | 2.7 | | 3.6 | 7.8-11.4 | | >40 | 100 |
|  | SAN | n.i. | | 1.4 | | n.i. | 2.9 | | n.i. | 7.9-10.3 | | >30 | 170 |
|  | PAN | n.i. | | 1.5 | | n.i. | 3.7 | | n.i. | 7.9-11.2 | | >40 | 50 |
|  |  |  | |  | |  |  | |  |  | |  |  |
| VCI-8 | LAN | n.i. | | n.i. | | n.i. | 3.3 | | n.i. | 8.6-12.5 | | >30 | 90 |
|  | SAN | n.i. | | n.i. | | 1.7 | 3.7 | | n.i. | 8.9-12.5 | | >30 | 80 |
|  | PAN | n.i. | | n.i. | | n.i. | n.i. | | n.i. | 9.5-11.9 | | >30 | 50 |
|  |  |  | |  | |  |  | |  |  | |  |  |
| VCI-9 | LAN | n.i. | | 1.5 | | 2.4 | 3.7 | | n.i. | 8.2-11.3 | | >40 | 100 |
|  | SAN | n.i. | | 1.8 | | 2.7 | 4.5 | | n.i. | 8.8-11.8 | | >40 | 150 |
|  | PAN | n.i. | | 1.7 | | n.i. | 3.0 | | 4.9 | 7.3-10.2 | | >40 | 100 |

Table 2: Overview of short latency measurements VCI-5 (n.i. = not identifiable).

|  | Stim amp (cu) | I  (ms) | II  (ms) | III  (ms) | IV  (ms) | p  (ms) | III  ampl.  (uV) | III SNR | p-n  amp.  (uV) | p-n  SNR | Repro-  ducibility (%) | Resid. Noise (nV) |
| --- | --- | --- | --- | --- | --- | --- | --- | --- | --- | --- | --- | --- |
| LAN | 50 | n.i. | n.i. | n.i. | n.i. | n.i. | n.i. | n.i. | n.i. | n.i. | 36 | 45 |
|  | 75 | 1.1 | 1.5 | 1.9 | 2.9 | 8.9 | .16 | 3.6 | .32 | 7.1 | 43 | 46 |
|  | 100 | 1.1 | 1.3 | 1.7 | 2.8 | 7.2 | .34 | 6.1 | 6.3 | 113 | 95 | 55 |
|  | 150 | 1.1 | 1.4 | 1.7 | 2.7 | 7.0 | .53 | 9.5 | 4.0 | 71 | 95 | 56 |
|  | 200 | 1.1 | 1.4 | 1.7 | 2.8 | 7.0 | .50 | 5.3 | 5.4 | 57 | 95 | 94 |
|  |  |  |  |  |  |  |  |  |  |  |  |  |
| SAN | 50 | n.i. | n.i. | n.i. | n.i. | n.i. | n.i. | n.i. | n.i. | n.i. | 0 | 32 |
|  | 75 | 1.1 | 1.3 | 1.7 | 2.9 | 7.2 | .53 | 11 | 1.1 | 24 | 62 | 45 |
|  | 100 | 1.1 | 1.3 | 1.9 | 3.0 | 7.1 | .36 | 9.7 | 1.4 | 38 | 77 | 37 |
|  | 150 | 1.1 | 1.3 | 1.7 | 2.9 | 7.4 | .50 | 9.6 | 2.4 | 45 | 95 | 52 |
|  | 200 | 1.1 | 1.3 | 1.7 | 2.9 | 7.5 | .52 | 14 | 4.1 | 107 | 99 | 38 |
|  |  |  |  |  |  |  |  |  |  |  |  |  |
| PAN | 50 | n.i. | n.i. | n.i. | n.i. | n.i. | n.i. | n.i. | n.i. | n.i. | 0 | 85 |
|  | 100 | n.i. | n.i. | n.i. | n.i. | 10.5 | n.i. | n.i. | .51 | 10 | 0 | 51 |
|  | 150 | 1.3 | 1.7 | 2.1 | 3.1 | 10.7 | .32 | 8.2 | .70 | 18 | 20 | 39 |
|  | 175 | 1.3 | 1.7 | 1.5 | 3.1 | 7.9 | .31 | 5.7 | 1.1 | 20 | 75 | 54 |
|  | 200 | 1.1 | 1.3 | 1.7 | 3.1 | 7.4 | .40 | 8.2 | 2.3 | 47 | 100 | 49 |
|  | 250 | 1.1 | NA | 1.7 | 2.7 | 7.1 | .41 | 1.4 | 8.8 | 30 | 99 | 288 |
| Mean (SD) | | 1.1  (.1) | 1.41 (.16) | 1.75  (.15) | 2.91  (.14) | 7.92  (1.30) | 0.41  (0.11) |  |  |  |  |  |

Table 3: Overview of EEG measurements on electrode CCP2h. VCI-8 LAN 50% UCL was not identifiable as no response was visible in the recording. VCI-9 50% UCL was not available as the UCL was close to the threshold of perception.

|  |  | Stim. amp. 50% UCL | | |  | Stim. amp. 100% (UCL) | | |
| --- | --- | --- | --- | --- | --- | --- | --- | --- |
| Participant | Electrode | n  (ms) | p  (ms) | n-p amp.  (µv) |  | n  (ms) | p  (ms) | n-p  amp.  (µv) |
| VCI-5 | LAN | 119 | 287 | 1.54 |  | 63 | 262 | 4.66 |
|  | SAN | 64 | 320 | 0.56 |  | 68 | 283 | 3.51 |
|  | PAN | 59 | 295 | 3.52 |  | 94 | 285 | 2.33 |
|  |  |  |  |  |  |  |  |  |
| VCI-8 | LAN | Not identifiable | | |  | 92 | 205 | 1.17 |
|  | SAN | 143 | 236 | 1.38 |  | 113 | 195 | 1.40 |
|  | PAN | 125 | 205 | 1.18 |  | 88 | 199 | 1.50 |
|  |  |  |  |  |  |  |  |  |
| VCI-9 | LAN | Not available | | |  | 172 | 244 | 1.29 |
|  | SAN |  |  |  |  | 187 | 338 | 1.15 |
|  | PAN |  |  |  |  | 154 | 240 | 0.72 |

*Figure 1: Impression of evoked potentials from* participants VCI-5 (orange), *VCI-2 (red), VCI-7 (purple), VCI-8 (yellow) and VCI-9 (blue). VCI-1 and VCI-6 recordings had very high residual noise levels and are therefore not visualized. Left: 1-5 ms responses; right: 1-24 ms responses.*

*Figure 2: electrically evoked responses of VCI-5 as a result of cochlear implant stimulation. Both figures show the three same responses with left the 1-7 ms interval and right 1-13 ms.*
